# Assessing resource allocation based on workload: a data envelopment analysis study on clinical departments in a class a tertiary public hospital in China

**DOI:** 10.1186/s12913-023-09803-y

**Published:** 2023-07-28

**Authors:** Xiaoxiong Hao, Lei Han, Danyang Zheng, Xiaozhi Jin, Chenguang Li, Lvshuai Huang, Zhaohui Huang

**Affiliations:** 1 Department of Health Service, General Hospital of Central Theater Command, Wuhan, 430070 People’s Republic of China; 2 Department of Critical Care Medicine, General Hospital of Central Theater Command, Wuhan, 430070 People’s Republic of China; 3 Department of Health Service, Medical Training Base, Army Medical University, Chongqing, 400038 People’s Republic of China; 4 General Hospital of Central Theater Command, Postdoctoral Workstation, Wuhan, 430070 People’s Republic of China

**Keywords:** Public hospitals, Resource allocation, Efficiency, K-means, Data envelopment analysis

## Abstract

**Objective:**

Today, the development mode of public hospitals in China is turning from expansion to efficiency, and the management mode is turning from extensive to refined. This study aims to evaluate the efficiency of clinical departments in a Chinese class A tertiary public hospital (Hospital M) to analyze the allocation of hospital resources among these departments providing a reference for the hospital management.

**Methods:**

The hospitalization data of inpatients from 32 clinical departments of Hospital M in 2021 are extracted from the hospital information system (HIS), and a dataset containing 38,147 inpatients is got using stratified sampling. Considering the non-homogeneity of clinical departments, the 38,147 patients are clustered using the K-means algorithm based on workload-related data labels including *inpatient days, intensive care workload index, nursing workload index, and operation workload index*, so that the medical resource consumption of inpatients from non-homogeneous clinical departments can be transformed into the homogeneous workload of medical staff. Taking the numbers of doctors, nurses, and beds as input indicators, and the numbers of inpatients assigned to certain clusters as output indicators, an input-oriented BCC model is built named the *workload-based DEA model*. Meanwhile, a control DEA model with the *number of inpatients* and *medical revenue* as output indicators is built, and the outputs of the two models are compared and analyzed.

**Results:**

Clustering of 38,147 patients into 3 categories is of better interpretability. 14 departments reach DEA efficient in the workload-based DEA model, 10 reach DEA efficient in the control DEA model, and 8 reach DEA efficient in both models. The workload-based DEA model gives a relatively rational judge on the increase of income brought by scale expansion, and evaluates some special departments like Critical Care Medicine Dept., Geriatrics Dept. and Rehabilitation Medicine Dept. more properly, which better adapts to the functional orientation of public hospitals in China.

**Conclusion:**

The design of evaluating the efficiency of non-homogeneous clinical departments with the workload as output proposed in this study is feasible, and provides a new idea to quantify professional medical human resources, which is of practical significance for public hospitals to optimize the layout of resources, to provide real-time guidance on manpower grouping strategies, and to estimate the expected output reasonably.

**Supplementary Information:**

The online version contains supplementary material available at 10.1186/s12913-023-09803-y.

## Introduction

### Development of public hospitals in China

Public hospitals are the main body of the medical service system in China, and the reform and development of public hospitals is an important part of Chinese healthcare reform. In August 2018, the National Health Commission put forward the concept of *high-quality development* for the first time [1]. In January 2019, The General Office of the State Council pointed out that improving the efficiency of management is the key to promoting the high-quality development of public hospitals [2]. In June 2021, The General Office of the State Council systematically elaborated the core of high-quality development of public hospitals, i.e., the development mode turns from expansion to efficiency, the management mode turns from extensive to refined, and the resource allocation mode turns from focusing on material elements to paying more attention to talent and technology elements. In September 2021, the National Health Commission proposed action for improving hospital management and took data-based decision support as an important method. It can be seen that the country's understanding of the high-quality development of public hospitals has gone through 3 stages: putting forward the concept, enriching the content, and focusing on the action. Today, refined management focusing on the allocation of medical resources is just the action to promote the high-quality development of public hospitals.

### Studies on medical resource allocation

In recent years, researchers have carried out fruitful studies on refining and optimizing medical resource allocation management. According to the methods used, these studies can be roughly divided into 3 categories: studies based on survey analysis, studies based on the model algorithm, and studies based on the computer system.

*Studies based on survey analysis* give more importance to reflecting on the current situation and putting forward suggestions on adjustment and optimization. To get conclusions, these studies are mainly carried out by collecting objective data and using basic mathematical or statistical methods, such as investigation, retrospective longitudinal study, cross-sectional prospective study, expert interview, semi-structured interview, and systematic review. The conclusions of these studies involve long shifts in hospitals, human resource management strategies on the medical staff, the workload of medical staff, patient-nurse ratio, balance of medical resource distribution, and so on [3–9].

*Studies based on the model algorithm* give more importance to proposing a new model or improving an existing algorithm. Using the results of the models and algorithms, these studies can provide suggestions for optimizing hospital management and resource allocation. Because of the large number of available models and algorithms, this category is also the most reported one. Models or algorithms used in these studies include robust optimization algorithms, stochastic optimization models, structural equation models (SEM), mixed integer linear programming (MILP), neural network model, Markov queuing model, data envelopment analysis (DEA), etc. Application scenarios of these models and algorithms include: determining the surgical schedule, balancing resource utilization and cost of service, minimizing hospital stays and medical resource waste, predicting patient flows, adjusting the distribution of resources, improving resource utilization and patient satisfaction, etc. [10–18].

*Studies based on the computer system* give more importance to the application of relatively mature system platforms to achieve the function of decision support, but the specific process of system development and related algorithms are usually not elaborated in detail. Computer systems used in these studies can be software tools, a simulation system, or a productivity dashboard based on HIS. And these computer systems can achieve medical data visualization, realistic scene simulation, project rapid evaluation, etc. [19–22].

### Applications of DEA in healthcare

Among the models and algorithms that can be used to optimize hospital management and resource allocation, the DEA model has a concise computational process, interpretability of model conclusions, and the ability to quantitatively evaluate the efficiencies of multiple units based on multiple indicators. In recent years, researchers paid more and more attention to the application of the DEA model in the field of healthcare. Wang X et al. used the DEA method to evaluate the operating efficiency of maternal and child health hospitals in a province in China, and assessed the allocation of health resources in poverty and non-poverty county by comparing the operating efficiency [23]. Vrabková I et al. applied the DEA model to evaluate the operation of 47 public hospitals in the Czech Republic, and put forward suggestions to optimize human resources and nursing quality [24]. In addition, as reported by Antunes BBP et al., the DEA model provided managers with the information needed to identify not only the outcomes to be achieved but the levels of resources needed to provide efficient healthcare [18].

It should be especially noted that an important prerequisite for DEA is that the DMUs to be evaluated should meet the homogeneity requirement, which is particularly important for the application of the DEA model to the healthcare field because hospitals, departments, and personnel with different professional backgrounds are non-homogeneous DMUs. Some researchers treated them as homogeneous DMUs and got certain results, which are not rigorous enough. Other researchers made some attempts to solve the problem of DEA with non-homogeneous DMUs, and the main idea of these attempts is to divide the non-homogeneous DMUs into several groups of homogeneous DMUs and then conduct DEA in groups. Cinaroglu S et al. combined the K-means clustering algorithm with DEA to study the technical efficiency of Turkish public hospitals. In the study, 81 provinces in Turkey were first clustered into 5 groups, and then DEA was conducted for hospitals within each group [25]. Hashem Omrani et al. combined fuzzy clustering, DEA, and cooperative gaming in their study on Iranian hospitals, first clustering 288 hospitals by the fuzzy C-means method and then conducting DEA for hospitals in each cluster [26]. In a study of 37 hospitals in Hong Kong, Li Y et al. first clustered hospitals into homogenous groups and divided the operation process of hospitals into several subunits. By DEA on the subunits of hospitals within the homogenous group, the efficiency of hospitals was measured [27].

The main purpose of this study is to provide a scientific basis for further adjustment and optimization of medical manpower and bed resource allocation in Hospital M, a Class A Tertiary Public Hospital in China. To complete this, the DEA study is conducted on all clinical departments of Hospital M, and operational efficiency and resource input redundancy are analyzed. Hospital M is located in Wuhan, Hubei Province, China. The hospital has long served society and strictly controlled the scale of expansion. With the promoting of reform in national public hospitals, Hospital M urgently needs to improve quality and efficiency. Considering the non-homogeneity among different clinical departments, we introduce proper assessment variables based on workload and applicable to non-homogeneous clinical departments. The differences in assessment results between these variables and commonly used variables are compared and analyzed, and the advantages and matters needing attention of applying these variables are discussed.

## Methods

### DEA model

In 1978, Charnes A et al. applied the data envelopment analysis (DEA) method for the first time to evaluate the relative effectiveness of homogeneous departments with multiple input and output indicators [28]. According to a review by Emrouznejad A, DEA had been widely used in agriculture, banking, supply chain, transportation, public policy, etc. by 2016 [29].

In a DEA model, the units to be evaluated are called decision making units (DMUs). DMUs can be large-scale administrative regions or certain organizations, such as provinces, cities, companies, universities, hospitals, etc. Chu C C et al. conducted a DEA study, in which East China, South China, North China, Central China, Northeast China, Southwest China, and Northwest China regions were taken as DMUs [30]. Seddighi H et al. conducted a DEA study and took 10 branches of the Iranian Red Crescent Society as DMUs [31]. As the study progressed, some smaller-scale DMUs including individuals also can be studied by the DEA model, which indicated the possibility of using the DEA model to guide micro-management. Vafaee Najar A et al. took individual nursing staff as DMUs and conducted a DEA study on 30 nurses in the cardiology department of a hospital in Iran [32].

The production possibility set describes the mapping relationship between the inputs and outputs of DMUs. It can be represented as.

$$\mathrm T=\left\{\left(\mathrm\,X,\mathrm Y\right)\vert\mathrm Y\geq0\,\mathrm{can\,be\,produced\,from\,X}\geq0\right\}.$$

There are certain postulates containing Convexity, Inefficiency, Ray Unboundedness, Minimum Extrapolation, etc. [33]. Combinations of postulates indicate various production possibility sets, forming 4 classic DEA models: *CCR model*, proposed by Charnes A, Cooper WW, Rhodes E in 1978 [28]; *BCC model*, proposed by Banker R D, Charnes A, Cooper W W in 1984 [33]; *FG model*, proposed by Färe R, Grosskopf S in 1985 [34]; *ST model*, proposed by Seiford L M, Thrall R M in 1990 [35]. All of the 4 models can be solved by linear programming model (LPM) with similar expressions.

The input-oriented DEA model can be expressed as follow.

$$\mathrm{min \theta }$$

$$\mathrm{s}.\mathrm{t}. \sum_{\mathrm{j}=1}^{\mathrm{n}}{\uplambda }_{\mathrm{j}}{\mathrm{x}}_{\mathrm{ij}}\le\uptheta {\mathrm{x}}_{\mathrm{ik}}$$

$$\sum_{\mathrm{j}=1}^{\mathrm{n}}{\uplambda }_{\mathrm{j}}{\mathrm{y}}_{\mathrm{rj}}\ge {\mathrm{y}}_{\mathrm{rk}}$$

$$\sum_{\mathrm{j}=1}^{\mathrm{n}}{\uplambda }_{\mathrm{j}}\ge 0\left(\mathrm{CCR}\right)\mathrm{ or }\sum_{\mathrm{j}=1}^{\mathrm{n}}{\uplambda }_{\mathrm{j}}= 1\left(\mathrm{BCC}\right)\mathrm{ or }\sum_{\mathrm{j}=1}^{\mathrm{n}}{\uplambda }_{\mathrm{j}}\le 1\left(\mathrm{FG}\right)\mathrm{ or }\sum_{\mathrm{j}=1}^{\mathrm{n}}{\uplambda }_{\mathrm{j}}\ge 1(\mathrm{ST})$$

$$\uplambda \ge 0$$

$$\mathrm{i}=\mathrm{1,2},\dots ,\mathrm{m};\mathrm{ r}=\mathrm{1,2},\dots ,\mathrm{q j}=\mathrm{1,2},\dots ,\mathrm{n}$$

The output-oriented DEA model can be expressed as follow.

$$\mathrm{max \varphi }$$

$$\mathrm{s}.\mathrm{t}. \sum_{\mathrm{j}=1}^{\mathrm{n}}{\uplambda }_{\mathrm{j}}{\mathrm{x}}_{\mathrm{ij}}\le {\mathrm{x}}_{\mathrm{ik}}$$

$$\sum_{\mathrm{j}=1}^{\mathrm{n}}{\uplambda }_{\mathrm{j}}{\mathrm{y}}_{\mathrm{rj}}\ge \mathrm{\varphi }{\mathrm{y}}_{\mathrm{rk}}$$

$$\sum_{\mathrm{j}=1}^{\mathrm{n}}{\uplambda }_{\mathrm{j}}\ge 0\left(\mathrm{CCR}\right)\mathrm{ or }\sum_{\mathrm{j}=1}^{\mathrm{n}}{\uplambda }_{\mathrm{j}}=1\left(\mathrm{BCC}\right)\mathrm{ or }\sum_{\mathrm{j}=1}^{\mathrm{n}}{\uplambda }_{\mathrm{j}}\le 1\left(\mathrm{FG}\right)\mathrm{ or }\sum_{\mathrm{j}=1}^{\mathrm{n}}{\uplambda }_{\mathrm{j}}\ge 1(\mathrm{ST})$$

$$\uplambda \ge 0$$

$$\mathrm{i}=\mathrm{1,2},\dots ,\mathrm{m};\mathrm{ r}=\mathrm{1,2},\dots ,\mathrm{q j}=\mathrm{1,2},\dots ,\mathrm{n}$$

Here $m$ is the number of inputs, $q$ is the number of outputs, $n$ is the number of DMUs.

### DMUs

In this study, all 32 clinical departments of Hospital M were taken as DMUs, including: Department of Emergency, Department of Rehabilitation Medicine, Department of Pediatrics, Department of Stomatology, Department of Obstetrics and Gynecology, Department of Geriatrics I, Department of Cardiothoracic Surgery, Department of Geriatrics II, Department of Geriatrics III, Department of Critical Care Medicine, Department of Oncology, Department of Hemodialysis, Department of Dermatology, Department of Nephrology, Department of Hematology, Department of Gastroenterology, Department of Infection, VIP Ward, Department of Neurology, Department of Special Medicine, Traditional Chinese Medicine Department (TCM), Department of General Practice, Department of General Surgery, Department of Neurosurgery, Department of Endocrinology, Department of Respiratory Medicine, Department of Cardiovascular Medicine, Department of Urology, Department of Ophthalmology, ENT, Department of Rheumatology and Immunology, Department of Orthopedics.

### Input indicators

Imani A et al. reviewed 144 research reports from 2010 to 2019 and systematically sorted out factors that could affect hospital efficiency, dividing them into input indicators, process indicators, and output indicators, which provides a good reference [36]. Manpower, beds, money, and equipment are input indicators commonly used in DEA studies in the field of hospital management. Meanwhile, according to the findings of Golany B et al. and Dyson R G et al., the number of indicators in a DEA model should not exceed half of the number of DMUs, and the product of the number of input indicators and the number of output indicators should not exceed half of the number of DMUs [37, 38]. Considering the actual situation of Hospital M, we selected 5 representative indicators as input indicators of our DEA model (Table 1).

**Table 1:** Input indicators of the DEA model

No	Input indicators	Definition
I1	Number of bedside doctors	Number of doctors who directly perform medical service for inpatients, including physicians, attending doctors, technicians, technologists-in-charge
I2	Number of superior doctors	Number of doctors who perform medical service for inpatients by directing bedside doctors and administering relative complex operations, including chief physicians, associate chief physicians, directors, deputy directors, ward directors
I3	Number of bedside nurses	Number of nurses who directly perform medical care for inpatients, including assistant nurses, nurses, nurse practitioner, nurses-in- charge
I4	Number of superior nurses	Number of nurses who perform medical care for inpatients by directing bedside nurses and administering relatively complex operations, including chief nurses, deputy chief nurses, head nurses, ward head nurses
I5	Number of beds	Number of available beds

### Output indicators

The number of inpatients was the most commonly used indicator for hospital efficiency assessment [36]. In consideration of the differences in workload brought by different patients for medical staff, we split this most commonly used indicator as follows.

### Dataset building

Relying on the hospital information system (HIS) of Hospital M, information of inpatients in the whole year of 2021 was extracted to build a dataset. Hospital M was formed by the merger of 2 hospitals, and the HISs of the 2 hospitals were officially merged in 2020. Considering that the merged HIS was still unstable in 2020, and the outbreak of COVID-19 made a certain impact on the management of Hospital M, the representativeness of medical data in 2020 was not yet ideal. Therefore, we only extracted the data in 2021.

The original dataset D contained 64,592 inpatients and every inpatient had 13 workload-related data labels, including *inpatient days, critical days, serious days, ICU days, resuscitation times, special nursing days, grade 1 nursing days, grade 2 nursing days, grade 3 nursing days, time of level 1 operation (h), time of level 2 operation (h), time of level 3 operation (h), time of level 4 operation (h)*. Due to the limited data processing capability of the software, a secondary dataset d containing 38,147 patients was formed by a stratified sampling of patients admitted to Hospital M in the middle and early part of each month.

### Data label simplifying

To facilitate the subsequent data clustering, the 13 workload-related data labels needed to be simplified.

- (1) 4 intensive care workload-related data labels, namely, *critical days, serious days, ICU days, resuscitation times*, were merged into one data label, the *intensive care workload index*.
- (2) 4 nursing workload-related data labels, namely, *special nursing days, grade 1 nursing days, grade 2 nursing days, grade 3 nursing days*, were merged into one data label, the *nursing workload index*.
- (3) 4 surgical operation workload-related data labels, namely, *time of level 1 operation (h), time of level 2 operation (h), time of level 3 operation (h), time of level 4 operation (h)*, were merged into one data label, the *operation workload index*.

Finally, the 13 data labels were simplified to 4: *inpatient days, intensive care workload index, nursing workload index, and operation workload index.* The values of the 3 indexes were calculated using a simple weighted average method.

### K-means clustering

K-means is a classic algorithm for unsupervised machine learning, proposed in a research report by MacQueen J B et al. in 1965 [39]. After necessary data cleaning including outliers processing, missing values processing, and data standardization, 38,147 inpatients were clustered by the K-means algorithm based on the above 4 data labels. Parameter k was set ranging from 2 to 5, attempting to cluster 38,147 inpatients of Hospital M into 2, 3, 4, or 5 clusters and the results were presented in Table 2.

**Table 2:** Results of K-means clustering of in patients in Hospital M

k	Cluster	Number of inpatients in cluster	Operation workload index (Standardized)	Nursing workload index (Standardized)	Intensive care workload index (Standardized)	Inpatient days (Standardized)
2	1	34,144	0.011 ± 0.026	0.031 ± 0.019	0.003 ± 0.014	0.041 ± 0.023
	2	4003	0.055 ± 0.097	0.144 ± 0.082	0.022 ± 0.067	0.173 ± 0.087
3	1	32,070	0.009 ± 0.019	0.029 ± 0.017	0.003 ± 0.012	0.037 ± 0.019
	2	5401	0.054 ± 0.081	0.099 ± 0.039	0.015 ± 0.047	0.123 ± 0.039
	3	676	0.05 ± 0.115	0.274 ± 0.117	0.046 ± 0.11	0.315 ± 0.121
4	1	32,098	0.008 ± 0.017	0.029 ± 0.017	0.003 ± 0.011	0.038 ± 0.019
	2	3764	0.008 ± 0.02	0.116 ± 0.041	0.022 ± 0.058	0.14 ± 0.043
	3	1826	0.154 ± 0.081	0.074 ± 0.036	0.006 ± 0.021	0.097 ± 0.042
	4	459	0.054 ± 0.112	0.312 ± 0.121	0.047 ± 0.119	0.351 ± 0.131
5	1	24,164	0.007 ± 0.016	0.021 ± 0.011	0.002 ± 0.007	0.029 ± 0.012
	2	9618	0.012 ± 0.023	0.056 ± 0.017	0.008 ± 0.024	0.071 ± 0.019
	3	2421	0.007 ± 0.022	0.143 ± 0.044	0.026 ± 0.071	0.17 ± 0.044
	4	1631	0.163 ± 0.082	0.076 ± 0.038	0.007 ± 0.023	0.1 ± 0.044
	5	313	0.069 ± 0.128	0.35 ± 0.127	0.049 ± 0.119	0.393 ± 0.139

According to the actual working conditions of Hospital M and characteristics of the hospitalization data of the inpatients under 4 clustering schemes, we believed that clustering inpatients into 3 categories was more explanatory. Inpatients in Cluster 1 accounted for the majority, and the values of workload-related data labels were at the lowest level, indicating that nearly 85% of the inpatients admitted to Hospital M were patients with low medical resource consumption, and the workload of medical staff serving them was light. Inpatients in Cluster 2 accounted for about 14%, and the values of workload-related data labels, except the operation workload index, were at a moderate level, indicating that these inpatients were mainly surgical patients and the workload of medical staff serving them was medium. Inpatients in Cluster 3 accounted for the lowest proportion, only 1.77%, but the values of workload-related data labels were at the highest level, indicating that these inpatients were the patients with high medical resource consumption, and the workload of medical staff serving them was heavy.

Compared with clustering inpatients into 3 categories, clustering into 2 could not effectively distinguish between surgical inpatients and critically ill inpatients, and clustering 4 or 5 could not clearly define the clinical characteristics of inpatients in each cluster. Therefore, 3 output indicators of the DEA model were selected, namely, *number of inpatients in cluster 1, number of inpatients in cluster 2,* and *number of inpatients in cluster 3*. Meanwhile, we called the DEA model with these 3 indicators as output indicators *workload-based DEA model*.

### Control DEA model

For a DEA model, output indicators reflected the orientation of evaluation. Under the premise that the input indicators remained unchanged, another 2 output indicators commonly used including *number of inpatients* and *medical revenue* were selected to build a control DEA model to analyze the impact of different output indicators on DEA conclusions.

### Model and software

Among the 4 classic DEA models, CCR model is based on the assumption of constant returns to scale (CRS), and BCC model is based on the assumption of variable returns to scale (VRS). For a certain set of DMUs, CCR model has the largest production possibility set, BBC model has the smallest production possibility set, FG model and ST model are in between. In practice, CCR model and BCC model are relatively more used, and BCC model, improved based on CCR model, can not only obtain the technical efficiency (TE) that can be obtained by CCR model but also can further decompose TE into pure technical efficiency (PTE) and scale efficiency (SE).

In addition, according to the purpose of research, DEA models can be divided into two types: input-oriented DEA models emphasize minimizing inputs for given outputs; output-oriented DEA models emphasize maximizing outputs for given inputs. Considering that medical resource allocation belongs to the input issue, we selected the classic input-oriented BCC model in this study and used SPSS-PRO software to solve the DEA models.

## Results

### Inputs and outputs

The inputs and outputs of the 2 DEA models are presented in Table 3. For the workload-based DEA model, the input variables are got based on the statistics of manpower and bed resources of Hospital M in the middle of 2021, and the output variables are got based on the statistics of K-means clustering results. For the control DEA model, the input data are the same as the workload-based DEA model, and the output variables are replaced by the numbers of inpatients and medical revenues of the 32 clinical departments.

**Table 3:** Inputs and outputs of the 2 DEA models

Department/DMU	Inputs					Outputs			Outputs (control)	
	I1	I2	I3	I4	I5	O1	O2	O3	Oc1	Oc2
Emergency Dept.	37	3	108	5	23	412	23	37	472	13,279,442.37
TCM Dept.	10	4	13	1	37	321	37	1	359	4,844,941.24
Pediatrics Dept.	24	5	69	5	10	1821	10	5	1836	11,624,410
General Practice Dept.	7	1	6	1	3	16	3	0	19	198,400.46
Endocrinology Dept.	17	6	32	3	88	1910	88	3	2001	17,663,749.96
Stomatology Dept.	51	8	48	4	27	279	27	1	307	5,136,885
Respiratory Medicine Dept.	22	4	64	4	49	1302	49	78	1429	24,975,503.69
Obstetrics and Gynecology Dept.	56	7	93	6	69	1500	69	0	1569	22,732,618.28
Geriatrics Dept. I	9	3	44	3	111	25	111	30	166	12,858,615
Geriatrics Dept. II	12	3	41	4	408	6	408	9	423	19,410,707.58
Geriatrics Dept. III	6	2	27	1	206	61	206	38	305	10,593,602.07
Rehabilitation Medicine Dept.	65	7	36	3	379	1085	379	5	1469	21,746,636.48
Cardiothoracic Surgery Dept.	15	9	66	4	224	905	224	5	1134	31,956,225.95
Cardiovascular Medicine Dept.	30	7	84	4	74	2838	74	38	2950	38,901,695.63
Infection Dept.	12	5	29	3	52	958	52	8	1018	10,826,903.64
General Surgery Dept.	26	12	88	5	312	2287	312	6	2605	64,667,131.9
Urology Dept.	21	16	66	4	154	2240	154	1	2395	43,428,515.34
Gastroenterology Dept.	28	11	60	4	94	2148	94	20	2262	24,981,789.62
Special Medicine Dept.	3	2	0	1	1	49	1	0	50	464,009.87
Dermatology Dept.	16	2	16	2	4	174	4	1	179	866,339.02
Ophthalmology Dept.	35	8	57	3	18	2758	18	0	2776	27,241,289.91
Neurology Dept.	27	10	57	4	155	2140	155	39	2334	27,168,444.42
Neurosurgery Dept.	39	12	94	5	485	886	485	38	1409	89,945,267.49
VIP Ward	8	1	12	1	4	228	4	1	233	2,669,080.72
ENT Dept.	24	6	31	1	37	1518	37	0	1555	19,764,653.56
Nephrology Dept.	16	7	48	2	76	587	76	1	664	11,566,926.6
Oncology Dept.	36	7	48	4	174	1638	174	5	1817	25,852,931.18
Hemodialysis Dept.	15	3	59	2	33	281	33	4	318	3,208,658.01
Hematology Dept.	9	2	16	1	54	426	54	7	487	6,900,621.61
Critical Care Medicine Dept.	5	1	19	1	3	28	3	10	41	2,501,839.8
Rheumatology and Immunology Dept.	6	2	0	1	20	326	20	1	347	4,055,747.13
Orthopedics Dept.	45	16	99	7	756	2462	756	0	3218	97,907,573.89

I1: Number of bedside doctors; I2: Number of superior doctors; I3: Number of bedside nurses; I4: Number of superior nurses; I5: Number of beds; O1: Number of inpatients in Cluster 1; O2: Number of inpatients in Cluster 2; O3: Number of inpatients in Cluster 3; Oc1: Number of inpatients; Oc2: Medical revenue (¥)

### Results of efficiency

Efficiencies and returns-to-scale characteristics of the 32 clinical departments in the workload-based DEA model are presented in Table 4, and those in the control DEA model are presented in Table 5.

**Table 4:** Efficiencies and returns-to-scale characteristics of 32 clinical departments

Department/DMU	PTE	SE	TE	Type of scale inefficiency
Emergency Dept.	1	1	1	-
TCM Dept.	1	0.588	0.588	IRS
Pediatrics Dept.	0.932	0.963	0.898	IRS
General Practice Dept.	1	0.103	0.103	IRS
Endocrinology Dept.	1	1	1	-
Stomatology Dept.	0.658	0.726	0.478	IRS
Respiratory Medicine Dept.	1	1	1	-
Obstetrics and Gynecology Dept.	0.654	0.958	0.627	IRS
Geriatrics Dept. I	0.617	0.853	0.526	IRS
Geriatrics Dept. II	1	1	1	-
Geriatrics Dept. III	1	1	1	-
Rehabilitation Medicine Dept.	1	1	1	-
Cardiothoracic Surgery Dept.	0.862	0.993	0.856	DRS
Cardiovascular Medicine Dept.	1	1	1	-
Infection Dept.	0.862	0.912	0.786	IRS
General Surgery Dept.	1	0.993	0.993	DRS
Urology Dept.	1	1	1	-
Gastroenterology Dept.	0.898	0.981	0.881	DRS
Special Medicine Dept.	1	0.301	0.301	IRS
Dermatology Dept.	0.989	0.509	0.504	IRS
Ophthalmology Dept.	1	1	1	-
Neurology Dept.	1	1	1	-
Neurosurgery Dept.	1	0.751	0.751	DRS
VIP Ward	1	0.562	0.562	IRS
ENT Dept.	1	1	1	-
Nephrology Dept.	0.631	0.889	0.561	IRS
Oncology Dept.	0.846	0.995	0.842	DRS
Hemodialysis Dept.	0.539	0.655	0.353	IRS
Hematology Dept.	1	0.801	0.801	IRS
Critical Care Medicine Dept.	1	1	1	-
Rheumatology and Immunology Dept.	1	1	1	-
Orthopedics Dept.	1	1	1	-

*PTE* Pure technical efficiency, *SE* Scale efficiency, *TE* Technical efficiency, *IRS* Increasing return to scale, *DRS* decreasing return to scale

**Table 5:** Efficiencies and returns-to-scale characteristics of 32 clinical departments (control)

Department/DMU	PTE	SE	TE	Type of scale inefficiency
Emergency Dept.	0.933	0.945	0.882	IRS
TCM Dept.	1	0.501	0.501	IRS
Pediatrics Dept.	0.908	0.96	0.871	IRS
General Practice Dept.	1	0.065	0.065	IRS
Endocrinology Dept.	1	1	1	-
Stomatology Dept.	0.565	0.871	0.492	IRS
Respiratory Medicine Dept.	1	1	1	-
Obstetrics and Gynecology Dept.	0.684	0.964	0.659	IRS
Geriatrics Dept. I	0.769	0.795	0.612	IRS
Geriatrics Dept. II	1	0.889	0.889	IRS
Geriatrics Dept. III	1	0.81	0.81	IRS
Rehabilitation Medicine Dept.	0.792	0.995	0.788	IRS
Cardiothoracic Surgery Dept.	0.927	0.924	0.857	IRS
Cardiovascular Medicine Dept.	1	1	1	-
Infection Dept.	0.845	0.868	0.734	IRS
General Surgery Dept.	1	1	1	-
Urology Dept.	1	1	1	-
Gastroenterology Dept.	0.841	0.994	0.836	IRS
Special Medicine Dept.	1	0.288	0.288	IRS
Dermatology Dept.	0.969	0.455	0.441	IRS
Ophthalmology Dept.	1	1	1	-
Neurology Dept.	0.85	0.975	0.829	DRS
Neurosurgery Dept.	1	1	1	-
VIP Ward	1	0.553	0.553	IRS
ENT Dept.	1	1	1	-
Nephrology Dept.	0.671	0.787	0.528	IRS
Oncology Dept.	0.843	0.994	0.838	IRS
Hemodialysis Dept.	0.508	0.555	0.282	IRS
Hematology Dept.	1	0.735	0.735	IRS
Critical Care Medicine Dept.	1	0.699	0.699	IRS
Rheumatology and Immunology Dept.	1	1	1	-
Orthopedics Dept.	1	1	1	-

*PTE* Pure technical efficiency, *SE* scale efficiency, *TE* technical efficiency, *IRS* Increasing return to scale; *DRS* decreasing return to scale

It can be seen that, in the workload-based DEA model, 14 departments reach DEA efficient (TE = 1) including Emergency Dept., Endocrinology Dept., Respiratory Medicine Dept., Geriatrics Dept. II, Geriatrics Dept. III, Rehabilitation Medicine Dept., Cardiovascular Medicine Dept., Urology Dept., Ophthalmology Dept., Neurology Dept., ENT Dept., Critical Care Medicine Dept., Rheumatology and Immunology Dept., Orthopedics Dept. In the control DEA model, 10 departments reach DEA efficient including Endocrinology Dept., Respiratory Medicine Dept., Cardiovascular Medicine Dept., General Surgery Dept., Urology Dept., Ophthalmology Dept., Neurosurgery Dept., ENT Dept., Rheumatology and Immunology Dept., Orthopedics Dept. In both DEA models, 8 clinical departments achieve DEA efficient including Endocrinology Dept., Respiratory Medicine Dept., Cardiovascular Medicine Dept., Urology Dept., Ophthalmology Dept., ENT Dept., Rheumatology and Immunology Dept., Orthopedics Dept.

Meanwhile, in the workload-based DEA model, 13 departments are in the state of IRS and 5 departments are in the state of DRS. In the control DEA model, 21 departments are in the state of IRS and 1 department is in the state of DRS. Compared to the workload-based DEA model, the control DEA model tends to support further expansion of hospital scale. While expanding scale can certainly improve the economic indicators of hospitals, reckless expansion should not be the first selection for public hospital development, especially in the context of hospital development mode turning from expansion to efficiency. The workload-based DEA model properly evaluates the benefits brought by expansion, which is in line with the orientation of public hospital development at present.

### Results of input redundancy

Table 6 presents the input redundancy of manpower and bed resources in the workload-based DEA model, and Table 7 presents the input redundancy in the control DEA model. Comparing Table 6 and Table 7, we notice that the results of 6 departments including Emergency Dept., Rehabilitation Medicine Dept., Geriatrics Dept. I, II, and III, Critical Care Medicine Dept. differ greatly in the two DEA models. These departments reach DEA efficient in the workload-based DEA model (Geriatrics Dept. I does not reach DEA efficient, but the input redundancy is small), but do not reach DEA efficient in the control DEA model, and the input redundancy level is high.

**Table 6:** Input redundancy of 32 clinical departments in Hospital M

Department/DMU	I1	I2	I3	I4	I5
Emergency Dept.	0	0	0	0	0
TCM Dept.	0	0.83	0	0	0
Pediatrics Dept.	2.31	0	8.08	1.92	44.02
General Practice Dept.	0.44	0.04	0	0.06	0
Endocrinology Dept.	0	0	0	0	0
Stomatology Dept.	20.10	2.82	14.57	1.38	0
Respiratory Medicine Dept.	0	0	0	0	0
Obstetrics and Gynecology Dept.	16.18	0	16.59	1.46	0
Geriatrics Dept. I	0	0	1.84	0.79	1.58
Geriatrics Dept. II	0	0	0	0	0
Geriatrics Dept. III	0	0	0	0	0
Rehabilitation Medicine Dept.	0	0	0	0	0
Cardiothoracic Surgery Dept.	0	0	9.65	1.09	5.19
Cardiovascular Medicine Dept.	0	0	0	0	0
Infection Dept.	0	0.85	2.92	0.69	1.99
General Surgery Dept.	0	0.43	16.28	0.38	0
Urology Dept.	0	0	0	0	0
Gastroenterology Dept.	0	3.11	0	0.28	0
Special Medicine Dept.	0	0.30	0	0.15	0.75
Dermatology Dept.	5.34	0.45	2.73	0.74	0
Ophthalmology Dept.	0	0	0	0	0
Neurology Dept.	0	0	0	0	0
Neurosurgery Dept.	6.10	2.06	0	0	0
VIP Ward	2.09	0	0	0.24	0
ENT Dept.	0	0	0	0	0
Nephrology Dept.	0	1.63	7.12	0	0
Oncology Dept.	11.85	0	0	0	0
Hemodialysis Dept.	0.95	0	11.31	0.19	0
Hematology Dept.	0.25	0	0	0	0.23
Critical Care Medicine Dept.	0	0	0	0	0
Rheumatology and Immunology Dept.	0	0	0	0	0
Orthopedics Dept.	0	0	0	0	0

I1: Number of bedside doctors; I2: Number of superior doctors; I3: Number of bedside nurses; I4: Number of superior nurses; I5: Number of beds

**Table 7:** Input redundancy of 32 clinical departments in Hospital M (control)

Department/DMU	I1	I2	I3	I4	I5
Emergency Dept.	24.26	0.38	78.14	3.50	0
TCM Dept.	0	0.61	0	0	0
Pediatrics Dept.	2.24	0	7.84	1.87	42.69
General Practice Dept.	0.21	0.01	0	0.04	0
Endocrinology Dept.	0	0	0	0	0
Stomatology Dept.	20.63	2.83	15.49	1.54	0
Respiratory Medicine Dept.	0	0	0	0	0
Obstetrics and Gynecology Dept.	17.73	0	18.88	1.83	0
Geriatrics Dept. I	0	0	12.75	1.07	18.65
Geriatrics Dept. II	0.97	0	11.81	2.18	10.93
Geriatrics Dept. III	0	0	8.66	0.11	13.61
Rehabilitation Medicine Dept.	34.56	0	0	0	44.14
Cardiothoracic Surgery Dept.	0	1.78	13.05	0.96	15.02
Cardiovascular Medicine Dept.	0	0	0	0	0
Infection Dept.	0	0.05	2.23	0.66	0
General Surgery Dept.	0	0	0	0	0
Urology Dept.	0	0	0	0	0
Gastroenterology Dept.	0	2.91	0	0.33	0
Special Medicine Dept.	0	0.29	0	0.14	0.72
Dermatology Dept.	4.79	0.37	3.37	0.69	0
Ophthalmology Dept.	0	0	0	0	0
Neurology Dept.	0	0	0	0	1.25
Neurosurgery Dept.	0	0	0	0	0
VIP Ward	2.05	0	0	0.24	0
ENT Dept.	0	0	0	0	0
Nephrology Dept.	0	1.22	7.3	0.08	0
Oncology Dept.	11.97	0	0	0.45	0.22
Hemodialysis Dept.	0.56	0	9.03	0.18	0
Hematology Dept.	1.55	0	0	0	4.28
Critical Care Medicine Dept.	2.39	0.36	10.64	0.56	0
Rheumatology and Immunology Dept.	0	0	0	0	0
Orthopedics Dept.	0	0	0	0	0

I1: Number of bedside doctors; I2: Number of superior doctors; I3: Number of bedside nurses; I4: Number of superior nurses; I5: Number of beds

To explain the difference, we need to analyze the functions and characteristics of these departments. Emergency Dept. and Critical Care Medicine Dept. are indispensable clinical departments in public hospitals, and their characteristics are high work intensity and relatively low economic benefits, which are also objective reasons why a considerable number of doctors were reluctant to engage in these departments. Geriatrics Dept. I, II, and III are special departments for acute and chronic diseases of the old retired national cadres at different levels. These departments are part of the social welfare system and also an important form of social responsibility of public hospitals, which is particularly important in today's aging society in China [40]. Their characteristics are long-term hospitalization and relatively long-lasting human resource consumption. Rehabilitation Medicine Dept. of Hospital M is derived from Orthopedics Dept., and its function mainly focuses on postoperative rehabilitation of trauma patients including rehabilitation for special occupational groups, also undertaking certain social responsibility. Its characteristics are relatively long-lasting treatment and human resource consumption. These departments are important functional units for public hospitals to assume social responsibility, but are relatively less attractive to practitioners. Simply evaluating the efficiencies of these departments based on the number of patients admitted and revenue will further weaken their abilities, which is inconsistent with the development orientation of public hospitals at present. Reflecting healthcare outcomes by the workload of medical staff can give these special departments a relatively equitable evaluation.

## Discussion

### Selection of DEA models

This study is an attempt to use the DEA model to solve hospital micro-management problems and we select the classic BCC model as the key model. Note that the development of the DEA model itself is rapid. Up to now, in addition to the 4 classic DEA models, researchers have developed dozens of DEA models and gradually formed a DEA model system. To solve the problem of inconsistent weights in the evaluation process of different DMUs, the *cross-efficiency DEA model* was proposed by Sexton T R et al. in 1986 [41]. To solve the problem of multi-time evaluation of efficiency, the *Malmquist DEA model* was proposed by Färe R et al. in 1992 [42]. To solve the problem of efficiency comparison and ranking of efficient DMUs, the *super-efficiency DEA model* was proposed by Andersen P et al. in 1993 [43]. To reduce the impact of the extreme value of DMU efficiency on the results, the *bootstrap DEA model* was proposed by Simar L et al. in 1998 [44]. To further explain the evaluation process of DMUs, the *network DEA model* was proposed by Fare R et al. in 2000 [45]. To optimize the calculation of slack variables, the *SBM model* and *EBM model* were proposed by Tone K in 2001 and 2010 respectively [46, 47]. These models have been increasingly used in recent studies. Li NN et al. used a Malmquist DEA model to study the efficiency of county-level public hospitals in Anhui Province, China, and put forward improvement measures for the future development of hospitals [48]. Kim C et al. used a bootstrap DEA model to analyze the efficiency of players comprising the healthcare supply chain, seeking a way to optimize a healthcare supply chain [49]. Zhang T et al. used a dynamic network DEA model to study the productivity and healthcare efficiency of provincial capitals in China [50]. Gong G et al. used a network DEA model to evaluate the efficiency of the healthcare system in each province in China after healthcare reform and used Tobit regression to analyze the factors affecting the overall efficiency of the healthcare system in each province [51]. Hou Y et al. used a super-efficiency SBM model to measure the efficiency of secondary and tertiary hospitals and primary healthcare centers within the hierarchical medical system in China and made suggestions [52].

Considering the purpose of hospital management, we do not need to rank departments more scientifically and accurately, so the demand for the super-efficiency DEA model and cross-efficiency DEA model is relatively small. However, we expect that the model can provide more accurate suggestions for resource allocation adjustment, and there is room for improvement in the calculation of slack variables (input redundancy) in BCC model, a radial DEA model. In future work, we will consider the application of SBM model or EBM model. In addition, once accumulating certain interannual data, Malmquist model will also be used to assist decision-making, organizing, and controlling.

### Selection of output indicators

Selection of output indicators is the key to this study. According to the review by Imani A et al. [36], number of inpatients, number of discharged patients, medical revenue, surgery rate, resuscitation rate, mortality rate, etc. were often taken as output indicators in DEA models. However, in the context of further emphasis on the public welfare of public hospitals, economic indicators are no longer the key output indicators. Meanwhile, different clinical departments are non-homogenous DMUs, and some indicators have different baseline levels in different departments, which are not suitable to be directly used as output indicators. Considering that there are only 32 DMUs, the number of which is not large enough, it is not appropriate to use the idea of clustering DMUs like current research for reference.

Jiang M from the Health Development Research Center of the National Health Commission pointed out that the value of personnel is difficult to quantify, and the key is to quantify the healthcare outcomes of human resources [53]. Jiang’s opinion enlightens us to try to select output indicators from the perspective of personnel value. Although medical staff in different clinical departments have different professional backgrounds, they all transform their common value (time and energy) into healthcare behaviors by their professional knowledge and skills. According to the workload generated in this transformation process, the inpatients can be clustered and the numbers of inpatients in certain clusters are counted as the output variables. Compared with directly taking the total numbers of inpatients and medical revenues as the output variables, it can avoid the separation between the workload level and the number of inpatients admitted, as well as economic income caused by the professional differences among clinical departments. The results of our study also support the above considerations, and give a relatively reasonable evaluation of the departments with high workloads and low economic benefits.

### Resource allocation of Hospital M

In general, the allocation of medical manpower and bed resources in most clinical departments of Hospital M is relatively balanced. Even inefficient clinical departments, most of them have a relatively low resource input redundancy level. According to the results of the workload-based DEA model, 7 inefficient departments are with high input redundancy levels, including Pediatrics Dept., Stomatology Dept., Obstetrics and Gynecology Dept., Cardiothoracic Surgery Dept., General Surgery Dept., Oncology Dept., Hemodialysis Dept. Among these departments, Stomatology Dept. is a special one with more work fulfilled in outpatient instead of the ward, so the input redundancy of Stomatology Dept. is neglectable in this study. Besides, there are still 6 inefficient clinical departments. These departments will be the focus of the field research in the future. When other objective factors are excluded according to the actual situation, if there is still a high amount of input redundant in these departments, it is necessary to consider adjusting the resource allocation.

### Rational views on the results

To solve the problem of efficiency assessment among non-homogenous DMUs, we put forward the idea of using workload-related data labels to get the output indicators of the DEA model. It can be seen from the results that our idea is feasible, which distinguishes the efficiencies of 32 clinical departments in Hospital M and calculates the input redundancy. Comparing the workload-based DEA model with the control DEA model, we find that the results of the former better reflect the current requirements of society for public hospitals in China. This also can be referenced to carry out intra-hospital and inter-hospital efficiency evaluations, propose personnel incentive strategies, and optimize healthcare resource allocation.

It should be noted that, although workload, as a feedback outcome indicator, is of certain advantages compared with generally used indicators in the efficiency assessment of hospitals, it is not suitable to be used as a feedforward guiding indicator. The reason is that the quantification of workload is based on the consumed time and energy of the medical staff, and in the quantifying process, the treatment of the critically ill and high-level surgical operations can be equivalent to a certain length of hospitalization or nursing time. If the workload is taken as a guiding indicator, it may make doctors and nurses unilaterally improve the workload level by simply extending the length of hospitalization and nursing time, while avoiding relatively complicated professional medical operations. That is detrimental to the overall technical improvement of hospitals.

### Limitation

The 13 workload-related data labels selected in this study are mainly based on the experience of management team members in Hospital M. In the process of simplifying and merging data labels, we use a simple weighted average method, which to some extent, reduces the representativity of the data labels. In future work, we will consider the introduction of an expert system to select workload-related data labels more systematically using the Delphi method and use Analytic Hierarchy Process (AHP) method to assign more scientific weights to each data label to form a complete workload-related data label system of medical staff in clinical departments.

The results obtained by DEA are the relative effectiveness, which can only reflect the contrast of DMUs in a given scope. If the overall efficiency of all clinical departments in Hospital M is low or high, we still can’t accurately evaluate the efficiencies of these departments in a larger scope. At this point, it is necessary to introduce other groups of DMUs (such as clinical departments in other hospitals of the same level) for a comparative study, to evaluate the efficiencies of the clinical departments more objectively.

## Conclusion

Resource allocation and efficiency evaluation are the core issues of hospital management, and numbers of researches have been conducted using various models and algorithms. In this study, the classic BCC model is selected, and the non-homogeneous clinical departments, which are relatively micro and less involved in relevant studies, are taken as DMUs of the model. Data reflecting manpower, beds, and healthcare of inpatients in Hospital M in 2021 are used to assess the efficiencies of 32 clinical departments and medical resource allocation. In this study, we propose a new way to solve the non-homogenous DEA model, objectively present the distribution of workload on medical staff in a class A tertiary public hospital in China, and build a DEA model, the results of which are in line with the orientation of Chinese public hospital development at present. On the other hand, limited by energy and technical resources, there is still room for further improvement.

Today, the development mode of public hospitals is turning from expansion to efficiency, the management mode is turning from extensive to refined, and the resource allocation mode is turning from focusing on material elements to paying more attention to talent and technology elements. This study provides a new idea to quantify the human resources of hospitals from the perspective of operational workload, which is of practical significance for public hospitals to adjust the layout of resources, provide real-time guidance on hospital manpower grouping strategies, and estimate the expected output reasonably.

## Supplementary Information

**Additional file 1. **The supplementary material file is an excel named“data of inpatients 2021-38147”, containing the hospitalization data of 38,147inpatients from 32 clinical departments of Hospital M in 2021. All of the inpatientswere admitted to Hospital M in the middle and early part of every month. Thedata labels of these inpatients include *Admission time, Department, Totalcost, Cost of laboratory test, Cost of IMAGING,* and 13 workload-relatedones mentioned in our article, namely,* Inpatient days, Criticaldays, Serious days, ICU days, Resuscitation times, Special nursing days, Grade1 nursing days, Grade 2 nursing days, Grade 3 nursing days, Time of level 1operation (h), Time of level 2 operation (h), Time of level 3 operation (h), Timeof level 4 operation (h). *All of the data was extracted from the hospitalinformation system of Hospital M, and this process was implemented directlythrough background programming. The supplementary material file shows the rawstate of the extracted data.

## Data Availability

All data generated or analyzed during this study are included in this published article and its supplementary information files.

## Abbreviations

SEM: Structural equation models
MILP: Mixed integer linear programming
DEA: Data envelopment analysis
DMU: Decision making unit
LPM: Linear programming model
HIS: Hospital information system
CRS: Constant returns to scale
VRS: Variable returns to scale
TE: Technical efficiency
PTE: Pure technical efficiency
SE: Scale efficiency
TCM: Traditional Chinese Medicine Department
AHP: Analytic Hierarchy Process
